# Surgical strategy for lung transplantation in Kartagener syndrome

**DOI:** 10.1016/j.xjtc.2025.102179

**Published:** 2025-12-10

**Authors:** Naoki Date, Daisuke Nakajima, Ichiro Sakanoue, Hidenao Kayawake, Satona Tanaka, Hiroshi Date

**Affiliations:** Department of Thoracic Surgery, Kyoto University Graduate School of Medicine, Kyoto, Japan

**Figure:**
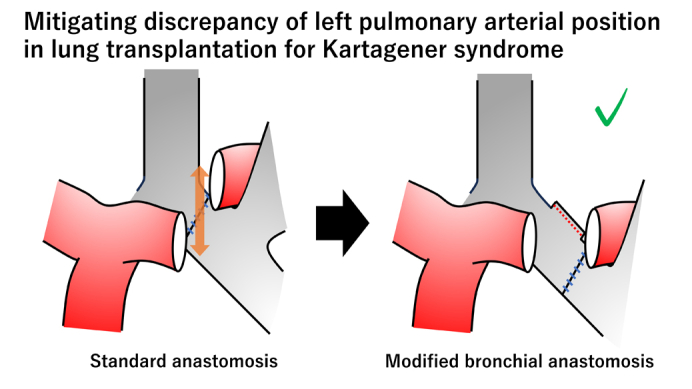
Modified bronchial anastomosis facilitates vascular anastomoses in Kartagener syndrome.

**Central Message**

Right middle lobectomy of the donor lung graft and bronchial anastomosis of the recipient's left bronchus intermedius enable safe lung transplantation in Kartagener syndrome.

Kartagener syndrome is a rare autosomal-recessive disorder characterized by the triad of situs inversus, bronchiectasis, and sinusitis. Lung transplantation in patients with Kartagener syndrome poses unique surgical challenges because of situs inversus. First, dextrocardia can compress the implanted right lung. In addition, reversed hilar structures complicate bronchus and pulmonary vessels anastomosis. In this study, we present our surgical strategy for lung transplantation in patients with Kartagener syndrome.

## Case Series

From January 2017 and December 2024, we performed bilateral lung transplantation in 4 patients with Kartagener syndrome. The cohort included 1 man (42 years) and 3 women (44, 49, and 57 years). All procedures used a clamshell thoracotomy under extracorporeal circulation (1 cardiopulmonary bypass and 3 extracorporeal membrane oxygenation cases). The institutional review board of Kyoto University Hospital approved this study on May 30, 2024 (reference number: R2389), and all patients provided written informed consent for the publication of study data.

**Figure 1:**
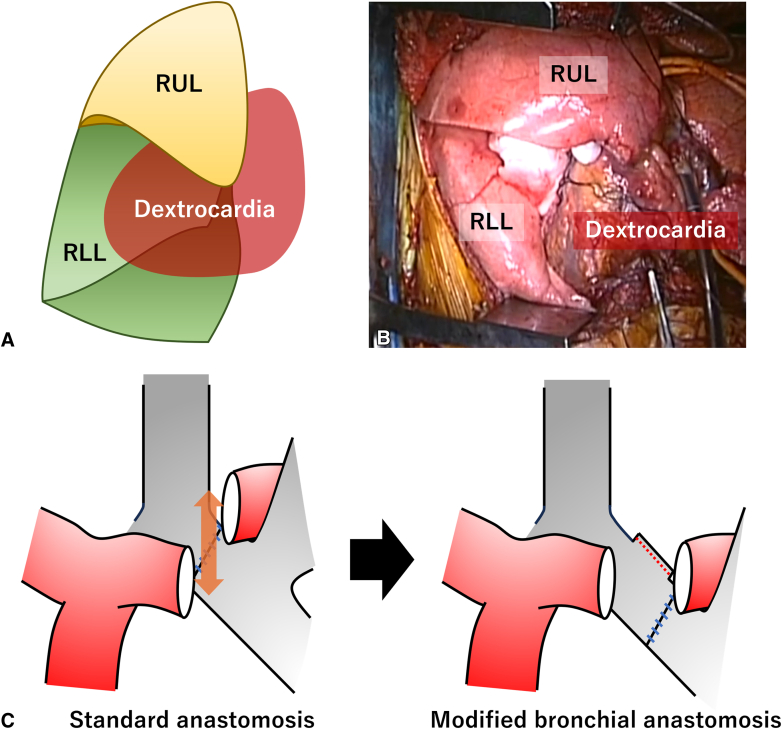
Schema (A) and picture (B) of middle lobectomy in the donor lung graft to accommodate dextrocardia. C, In the modified procedure, the donor left main bronchus was anastomosed to the recipient's left bronchus intermedius, which mitigated the positional discrepancy of the donor and recipient pulmonary arteries. *RUL*, Right upper lobe; *RLL*, right lower lobe.

The following surgical strategy was applied in all cases. First, during right lung transplantation, the donor's right middle lobe was resected on the back table, with the bronchial stump closed to accommodate the recipient's dextrocardia (Figure 1, *A* and *B*). Bronchial and vascular anastomoses were performed on the right side in the standard manner. Second, in left lung transplantation, the graft's left main bronchus was anastomosed to the recipient's left bronchus intermedius, with the recipient's left upper lobe bronchus closed. This modification shifted the hilar structures of the donor lung caudally, thereby minimizing the positional discrepancy between the donor and recipient pulmonary arteries (Figure 1, *C*). This allowed tension-free anastomoses of the left pulmonary artery and vein. No bronchial stump coverage was performed. After graft reperfusion, all patients were weaned from extracorporeal support intraoperatively in the usual manner (Video 1).

The operative and posttransplant outcomes are summarized in Table 1. Severe pleural adhesions were observed in all cases, resulting in prolonged operative time (564-721 minutes) and substantial blood loss (3160-17,000 mL), which was managed through continuous blood transfusion under careful monitoring of the bleeding volume. One patient required delayed chest closure on postoperative day 3. All patients were managed with a tracheostomy for frequent sputum suctioning and required prolonged postoperative mechanical ventilation (13-41 days). No bronchial or vascular anastomotic complications occurred. Regarding long-term survival, 1 patient died of chronic allograft dysfunction 6 years after transplantation, whereas 3 patients survived uneventfully for 1 year, 2 years, and 4 years.

**Table 1:** Operative and posttransplant outcomes of the patients

Case	Age/sex	Size matching, %∗	ECC	ECC time, min	Operative time, min	Blood loss, mL	Blood transfusion, mL	Complications/note	Postoperative ventilation, d	ICU stay, d	Hospital stay, d	Survival, y	Cause of death
1	44/F	28.3	CPB	260	564	8825	RBC: 1960 FFP: 2640 PLT: 800	Pleural effusion Pneumonia	13	9	102	6	CLAD
2	49/F	−5.2	ECMO	340	687	3340	RBC: 840 FFP: 1920 PLT: 200	Atelectasis of right lower lobe	37	11	63	>4	−
3	42/M	8.7	ECMO	463	721	3160	RBC: 280 FFP: 2640 PLT: 400	Pneumothorax	41	17	71	>2	−
4	57/F	−8.8	ECMO	290	598	17,000	RBC: 1960 FFP: 4650 PLT: 600	Delayed chest closure at POD3	13	20	63	>1	−

*ECC*, Extracorporeal circulation; *ICU*, intensive care unit; *F*, female; *CPB*, cardiopulmonary bypass; *RBC*, red blood cell; *FFP*, red blood cells; *PLT*, platelets; *CLAD*, chronic lung allograft dysfunction; *ECMO*, extracorporeal membrane oxygenation; *M*, male; *POD*, postoperative day.

∗ Size matching = (predicted donor vital capacity/recipient predicted vital capacity − 1) × 100. Based on the Japan Organ Transplant Network criteria, lung transplantation is eligible when the value is between −30% and +30%.

## Discussion

In patients with Kartagener syndrome, a small right chest cavity can cause donor right lung graft oversizing, which can lead to atelectasis of the implanted right lung graft.^1^ Although right lower-lobe resection has been reported as a downsizing strategy,^2^ we chose right middle-lobe resection. Given its anatomical location in the anteroinferior space of the right chest cavity, right middle lobectomy creates adequate space for the recipient's dextrocardia. In addition, various techniques, such as mobilization of the donor pulmonary artery or elongation of the vascular cuffs, have been reported to treat reverse hilar structures.^1^^,^^3^ Our approach uniquely featured bronchial anastomosis at the level of the recipient's left bronchus intermedius to adjust the position of the donor's left hilar vessels. This modified technique facilitates left pulmonary arterial and venous anastomoses without complications.

Our surgical strategy included leaving the bronchial stumps of the recipient's left upper-lobe bronchus and the donor's right middle-lobe bronchus closed. Potential concerns exist regarding bronchopleural fistulas; however, we believe that leaving bronchial stumps closed is considered acceptable. The donor's right middle bronchial stump may have the potential benefit of being covered by the remaining right upper and lower lobes, although there is no strong evidence that this reduces the risk of bronchopleural fistula compared with other types of lobectomies. Careful observation of the bronchial stump is required. We have reported that leaving recipient's bronchial stump is safe.^4^ The recipient's left upper bronchial stump healed well as a result of maintained bronchial arterial circulation. In conclusion, bilateral lung transplantation for Kartagener syndrome can be performed safely, with favorable outcomes using the presented surgical strategy.

## Conflict of Interest Statement

The authors reported no conflicts of interest.

The *Journal* policy requires editors and reviewers to disclose conflicts of interest and to decline handling or reviewing manuscripts for which they may have a conflict of interest. The editors and reviewers of this article have no conflicts of interest.

## Supplementary Data

Video 1 Operative video of bilateral lung transplantation for Kartagener syndrome. Video available at: https://www.jtcvs.org/article/S2666-2507(25)00560-7/fulltext.
