# Supplementary figures and images for: Surgical strategy for lung transplantation in Kartagener syndrome
Source: JTCVS Tech. 2025 Dec 10;35:102179. doi: 10.1016/j.xjtc.2025.102179 (PMC12881766; doi:10.1016/j.xjtc.2025.102179)

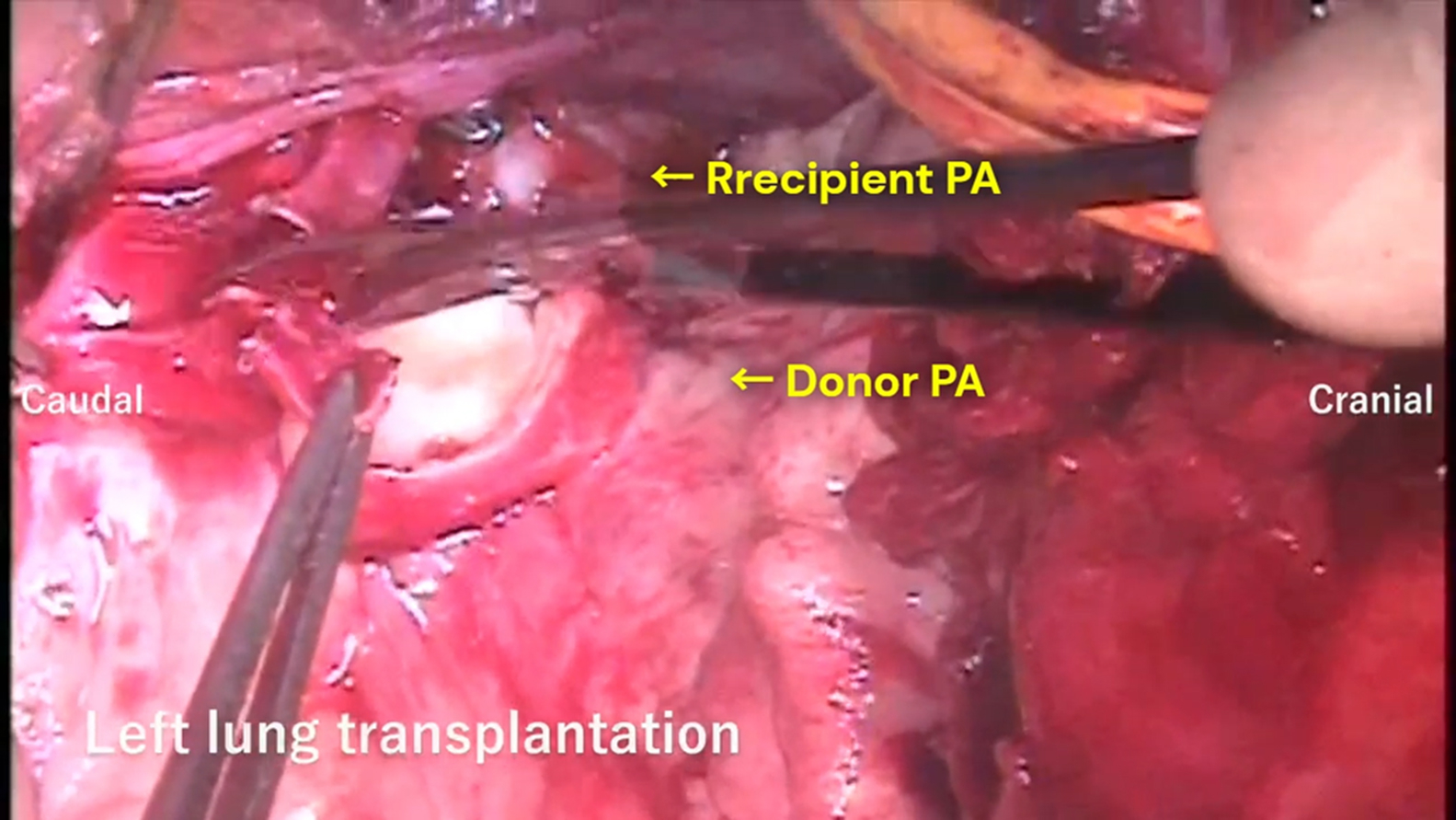

Supplement: Video 1 — Operative video of bilateral lung transplantation for Kartagener syndrome. Video available at: https://www.jtcvs.org/article/S2666-2507(25)00560-7/fulltext. [file fx2.jpg]
